# Full-band, multi-angle, multi-scale, and temporal dynamic field spectral measurements in China

**DOI:** 10.1038/s41597-023-02265-1

**Published:** 2023-06-03

**Authors:** Jianguang Wen, Xiaodan Wu, Qing Xiao, Qinhuo Liu, Mingguo Ma, Xingming Zheng, Yonghua Qu, Rui Jin, DongQin You, Yong Tang, Xingwen Lin, Wenpin Yu, Baochang Gong, Jian Yang, Yuan Han

**Affiliations:** 1 State Key Laboratory of Remote Sensing Science, Aerospace Information Research Institute, Chinese Academic of Sciences, Beijing, 100101 China; 2 University of Chinese Academic of Sciences, Beijing, 100049 China; 3 College of Earth and Environmental Sciences, Lanzhou University, Lanzhou, 730000 China; 4 Chongqing Jinfo Mountain Karst Ecosystem National Observation and Research Station, School of Geographical Sciences, Southwest University, Chongqing, 400715 China; 5 Northeast Institute of Geography and Agroecology, Chinese Academic of Sciences, Changchun, 130102 China; 6 Faculty of Geographical Science, Beijing Normal University, Beijing, 100875 China; 7 Northwest Institute of Eco-Environment and Resources, Chinese Academic of Sciences, Lanzhou, 730000 China; 8 College of Geography and Environmental Sciences, Zhejiang Normal University, Jinhua, 321004 China

**Keywords:** Environmental sciences, Biogeochemistry

## Abstract

Field-measured spectra are critical for remote sensing physical modelling, retrieval of structural, biophysical, and biochemical parameters, and other practical applications. We present a library of field spectra, which includes (1) portable field spectroradiometer measurements of vegetation, soil, and snow in the full-wave band, (2) multi-angle spectra measurements of desert vegetation, chernozems, and snow with consideration of the anisotropic reflectance of land surface, (3) multi-scale spectra measurements of leaf and canopy of different vegetation cover surfaces, and (4) continuous reflectance spectra time-series data revealing vegetation growth dynamics of maize, rice, wheat, rape, grassland, and so on. To the best of our knowledge, this library is unique in simultaneously providing full-band, multi-angle, multi-scale spectral measurements of the main surface elements of China covering a large spatial extent over a 10-year period. Furthermore, the 101 by 101 satellite pixels of Landsat ETM/OLI and MODIS surface reflectance centered around the field site were extracted, providing a vital linkage between ground measurements and satellite observations. The code language used for this work is Matlab 2016a.

## Background & Summary

Spectra characterize the distribution of electromagnetic radiation regimes reflected or emitted from the Earth’s surface along the spectral domains and demonstrate great potential in the remote sensing field^1^. Remote sensing algorithms detect target surfaces by using spectral information across different electromagnetic radiation regions, e.g., reflectivity in the solar reflective visible and near-infrared (VNIR), shortwave infrared (SWIR), emissivity in the thermal infrared (TIR), and reflectivity or emissivity in the microwave domains. Spectral features, such as peaks, valleys, magnitude, and derived spectral indices, are widely used in remote sensing spectral unmixing^2^, land cover mapping^3–6^, environmental monitoring^7–10^, among others. Spectra are also reserved as an initial boundary to parameterize the physical remote sensing models and constrain the subsequent parameter retrievals^11–13^. Moreover, they can be used as reference data to validate satellite observations. Therefore, accurate measurements of spectra will advance the modeling of remote sensing physical radiative transfer processes and thus improve large-scale monitoring and mapping of surface properties and dynamic changes.

Field-based spectra measurements provide observations under more controlled conditions compared to the measurements of satellites^14–21^. Great efforts have been made to collect the Earth surface spectra to build spectral libraries. Currently, a series of spectral libraries have been constructed to support science research and industry applications^22^. By and large, these libraries can be grouped into two categories: general spectral libraries and thematic spectral libraries. Libraries fall in the general category emphasize on the coverage of different land cover types and can serve for a variety of applications. Typical examples of these libraries include the United States Geological Survey (USGS) spectral library updated in 2017^23,24^, and the ASTER spectral library 2.0 updated to the ECOSTRESS spectral library 1.0^25^. By contrast, the latter category mainly serves the purpose for specific applications, mainly in realm of environmental monitoring. Examples of thematic libraries include the JPL (Jet Propulsion Laboratory) and the ASU (Arizona State University) mineral/rock spectral library^26^, the HyspIRI (Hyperspectral InfraRed Imager) vegetation ecosystem spectral library^27^, the ICRAF (International Centre for Research in Agroforestry) soil reflectance spectral library^28^, and the EcoSIS (ecological spectral information system) spectral library established in 2021 (https://ecosis.org/). These spectral libraries have been widely used in automated object identification routines or served for advanced automated data processing tasks.

However, there are several limitations to the exiting libraries. First, they provide spectra in shortwave, infrared, or microwave domains separately^29^. This discontinuity does not meet the requirements for developing a unified spectral-domain radiative transfer model and its associated applications^30–32^. Second, they rarely measure (and release) anisotropic spectra across different spectral domains. Nevertheless, apparent anisotropy exists in the surface reflectivity in the VNIR-SWIR spectral domain (e.g., bidirectional reflectance distribution function (BRDF))^33,34^, emissivity in the TIR spectral domain (e.g., directional bright temperature)^35,36^, and reflectivity or emissivity in the microwave spectral domains (e.g., directional microwave radiation)^37^. Ignoring the directionality of satellite observations may lead to large errors in the retrieval of remote sensing radiation and vegetation structure variables^38–41^. Therefore, the multi-angular spectra are a pressing need to advance the estimation of surface energy budget and modeling of land surface processes. Third, there is a lack of multiscale spectra measurements (e.g., leaf and canopy spectra), which hinders the understanding of cross-scale physical radiative transfer and associated ecological processes. Hence, the collection of multiscale spectra is much needed to help improve the understanding of cross-scale radiative transfer processes from individual leaves to canopies and even ecosystems and promote the exploration of scaling effects on ecological processes (e.g., upscaling plant function traits)^42,43^. Furthermore, the temporal dynamic spectra for typical vegetation are insufficient. Given the fact that the vegetation dynamics are crucial for modeling land cover change and plant phenology as well as its feedback to climate change^44–46^, vegetation spectra across different growth stages need to be further supplemented in order to improve the monitoring and modeling of land cover and land use change, and surface phenology in general.

In this paper, we present the full-wave band, multi-angle, multi-scale, and temporal dynamic spectral library dubbed Ground Object Spectral Library (GOSPEL). The full-band here refers to the spectral region from optical regime to microwave band, including optical reflection, infrared radiation, and microwave radiation. Multi-angle denotes the spectra measurements with different view angles using a multi-angle observation device. Multi-scale represents the spectral measurements at different spatial scales. Specifically, they include point scale (i.e., leaf scale), several meters scale (i.e., canopy scale), 30 m pixel scale, and 500 m pixel scale. Temporal dynamic spectra refer to the continuous field spectral measurements during the entire vegetation growing season. However, the observation frequency is not fixed. Instead, it depends on the speed of vegetation growth and the change of vegetation cover. To the best of our knowledge, this library is unique in simultaneously providing full-band, multi-angle, multi-scale, and temporal dynamic field spectral measurements of the main surface elements of China covering a large spatial extent, which is critical to have a complete understanding of the crucial influence factors on the spectral characteristics of ground objects. Here, the main surface elements refer to the several broad categories of land covers, including vegetation, soil, water, ice, snow, and artificial targets. All measurements correspond to a typical polar-orbiting satellite observation throughout the year with regards to solar zenith angle and close-to-nadir instrument viewing angle. Moreover, the 101 by 101 satellite pixels of Landsat ETM (Enhanced Thematic Mapper)/OLI (Operational Land Imager) surface reflectance (i.e., USGS Landsat Surface Reflectance Tier 1) and MODIS (Moderate-resolution Imaging Spectroradiometer) surface reflectance (i.e., MOD09GA/MYD09GA) centered around the field site were extracted, providing a vital linkage between ground measurements and satellite observations. We believe that this spectral library will undoubtedly advance the development of the modeling of plant growth and vegetation phenology, support further research on cross-scale ecological processes and underlying mechanisms, the quantification of the geometric effects of solar and sensor on the physical radiative transfer processes, and the retrieval of surface biophysical and biochemical parameters. Moreover, it can serve as the ground-truth information for validation of satellite spectral measurements.

## Methods

### The spatial distribution of measurement samples

**Fig. 1:**
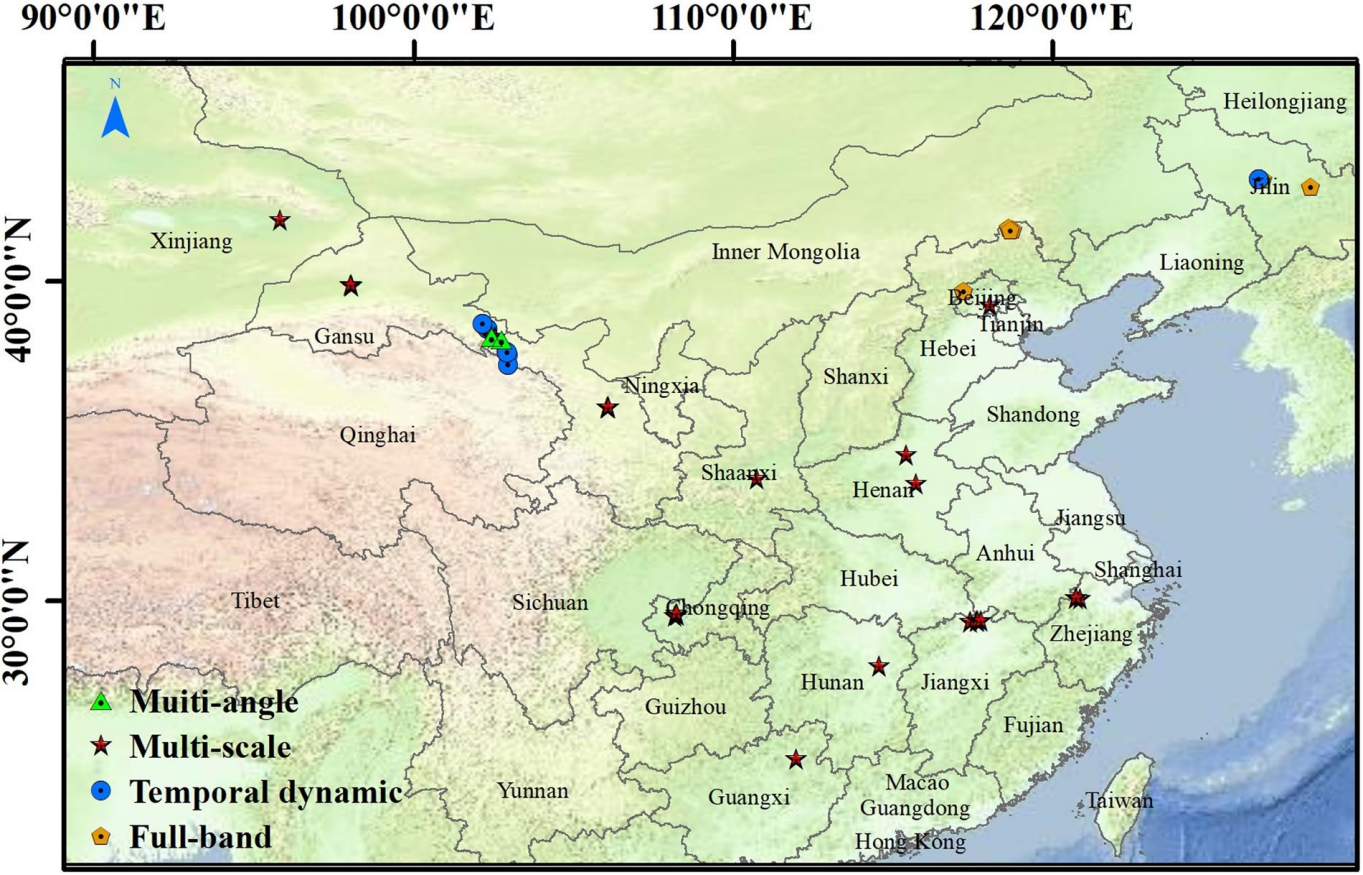
The locations of the main sites where most of the data records were measured.

The field measurements were made on the different land cover types over different areas in China (Fig. 1) over a period of 10 years. The spectroradiometer measurements in the full-band were made at three areas in North China (Chengde, Zhangjiakou, and Changchun). The multiangle spectra measurements were conducted in China’s northwest (Zhangye and Aletai) and northeast (Changchun). The multiscale reflectance spectra were collected over the main forest and crops in 16 locations covering various geographical regions of China (Guilin, Zhangye, Chongqing, Jiujiang, Baiyin, Xishuangbanna, Jiuquan, Hangzhou, Zhangjiakou, Lushan, Xi’an, Hami, Xinxiang, Changsha, Xuchang, and Beijing, refer to Fig. 1). The temporal dynamic field spectral measurements were carried out in typical agricultural areas of Changchun, Zhangye, and Haibei Tibetan Autonomous Prefecture. The measurement samples collected for this library represent a majority of land surface types in China. Each location of the field measurement site was at the centre of 101 by 101 satellite pixels such as Landsat ETM/OLI and MODIS surface reflectance. Each measurement plot is homogeneous in cover with about 2-km × 2-km field samples to ensure the spatial representativeness of the *in situ* measurements with respect to the satellite pixel^47^. In this way, data acquired at different spatial scales (the field measurements, the relatively high spatial resolution satellite/ airborne data, and the relatively coarse resolution satellite data) can all be linked to each other, thus providing favorable conditions for sensor design and validation of remote sensing products.

### The measurement geometry, quantities, and calibration

**Fig. 2:**
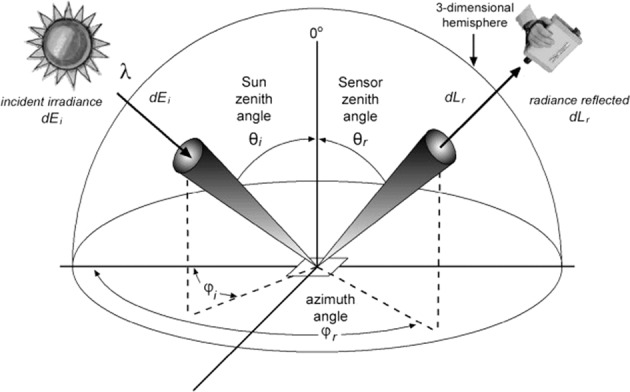
The general concept and parameters of BRDF (© copyright Steven *et al*.^65^, reprinted by permission of Informa UK Limited, trading as Taylor & Taylor & Francis Group). The target is bathed in solar irradiance (*dE*_*i*_) from a Sun zenith angle *θ*_*i*_ and azimuth angle *φ*_*i*_, and the sensor records the radiance (*dL*_*r*_) reflected by the target of interest at a specific zenith angle *θ*_*r*_ and azimuth angle *φ*_*r*_.

All spectral measurements correspond to the typical polar-orbiting satellite observations throughout the year regarding the solar zenith angle and close-to-nadir instrument viewing angle. The surface reflection exhibits an anisotropic behavior that is associated with illumination and viewing geometries. Objects generally look differently when viewed/ illuminated from different angles/ directions. This behavior shows a significant effect on higher-level surface biophysical properties such as vegetation indexes, LAI/FPAR, surface albedo, land cover, burned area, and land cover change. Fortunately, this phenomenon can be quantitatively described by BRDF (Bidirectional Reflectance Distribution Function) (Fig. 2).

The BRDF is mathematically expressed as the ratio of the target radiance *dL*_*r*_ reflected from the target in a specific viewing direction (*θ*_*r*_, *φ*_*r*_) to the Sun’s incident irradiance *dE*_*i*_ from the direction (*θ*_*1*_, *φ*_*i*_) (Eq. (1)). It is wavelength-dependent and is related to the structural and optical properties of the surface. Nevertheless, the BRDF cannot be measured directly since the amount of radiant flux is unmeasurable in the truly infinitesimal elements of a solid angle^48^. Therefore, a measurable quantity, namely BRF (Bidirectional Reflectance Factor), is generally used instead in the natural environment. The BRF is defined as the ratio of the target radiance *dL*_*r*_ reflected from the target in a specific viewing direction (*θ*_*r*_, *φ*_*r*_) to the radiance reflected from the white reference panel *dL*_*c*_ at the same illumination and view conditions (Eq. (2)). Ideally, the white reference panel should be highly reflective with a reflectance of 1 and exhibits Lambertian behavior in the visible and near-infrared region.

1 $$BRDF\left({\theta }_{i},{\varphi }_{i};{\theta }_{r},{\varphi }_{r};\lambda \right)=\frac{d{L}_{r}({\theta }_{r},{\varphi }_{r};\lambda )}{d{E}_{i}({\theta }_{i},{\varphi }_{i};\lambda )}$$

2 $$BRF=\frac{d{L}_{r}({\theta }_{r},{\varphi }_{r},\lambda )}{d{L}_{c}({\theta }_{r},{\varphi }_{r},\lambda )}$$

3 $$BRF=\frac{d{L}_{r}}{d{L}_{c}}=\frac{d{L}_{r}}{d{E}_{i}/\pi }=\pi \left(BRDF\right)$$

The BRF and BRDF can be mathematically related as shown in Eq. (3). Hence, it is the BRF that was measured in the field spectra measurements. In field measurements, the white reference panel was measured before each target observation and repeated when the illumination conditions changed during one measurement event. Theoretically, the target radiances can be converted to surface reflectance using Eq. (2). Nevertheless, the reflectance of the white reference panel is generally less than 1 in practice due to the panel degradation. Hence, the actual surface reflectance was calculated by calibrating the ratio of the target’s reflected radiation to the panel’s reflected radiation recorded by the spectroradiometer with the panel’s calibrated reflectance (Eq. (4)).

4 $$BRF=\frac{d{L}_{r}({\theta }_{r},{\varphi }_{r},\lambda )}{d{L}_{c}({\theta }_{r},{\varphi }_{r},\lambda )}{R}_{c}(\lambda )$$

where *R*_*c*_(*λ*) is the reflectance of the white reference panel which is calibrated in the laboratory.

The land surface emissivity with the wavelength of 8–14 um is defined as the ratio of the radiance emitted by a target and the radiance emitted by a black body at the same temperature. The field implementation of radiance from the blackbodies, sky, and target measurements by Hook and Kahle^49^ was followed. Specifically, the absolute spectral emissivity of the target ${\varepsilon }_{t}(\lambda )$ was calculated as:

5 $${\varepsilon }_{t}(\lambda )=\frac{{L}_{s}(\lambda )-{L}_{dwr}(\lambda )}{B(\lambda ,T)-{L}_{dwr}(\lambda )}$$

where *L*_*s*_(*λ*) represents the object radiation, *L*_*dwr*_(*λ*) denotes the downwelling calibrated radiation, and *B*(*λ*, *T*) is the radiation based on the Planck function at the same temperature. The linear emissivity constraint temperature and emissivity separation (LECTES) approach^50^ was employed to retrieve the emissivity and temperature from Eq. (5).

Microwave spectra include two kinds of radiation regime which are the microwave brightness temperature and microwave backscattering coefficient^51^. In this paper, we only focus on the microwave brightness temperature because it represents the outward radiation from the Earth itself. The brightness temperature measured by microwave radiometer (K) can be written as^52^:

6 $${T}_{B}=\frac{\int {\int }_{0}^{4\pi }TB(\theta ,\varphi )F(\theta ,\varphi )d\Omega }{\int {\int }_{0}^{4\pi }F(\theta ,\varphi )d\Omega }$$

where *TB*(*θ*, *φ*) denotes the brightness temperature radiated by a ground object in a solid angle at zenith angle (*θ*) and azimuth angle (*φ*). *F*(*θ, φ*) represents the antenna pattern, which describes the efficiency of the antenna in different observation directions. *d*Ω is the solid angle.

### The measurements setup

#### Full-band spectra measurements

**Fig. 3:**
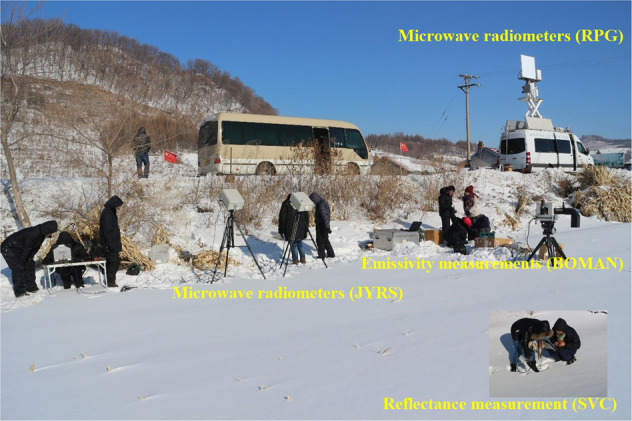
The full-band spectra (i.e., reflectance, thermal emissivity, microwave emission) measurements over a snow surface in Jiaohe of Jilin province.

Three spectrometer types were utilized to assemble the data records, namely the reflectance measurement from visible to shortwave infrared region (solar reflectance spectra), the emissivity measurement in the thermal infrared region (thermal emission spectra), and the microwave radiation measurement in the microwave region (microwave radiation). They were measured synchronously with ASD/SVC reflectance spectrometer, 102 F/BOMAN emissivity meter, and microwave radiometers (RPG/JYRS). Additional instruments were used to take measurements over snow cover, as shown in Fig. 3. All the spectral measurements are manually or vehicle-based implemented according to the height of the typical object in the experiments. The footprint of a single measurement depends on the distance between the sensor and the target as well as the measurement optics. The height of the sensor from the underlying surface has been made to be larger than 1 m. Since the field of view is different for different instruments, homogeneous areas were selected as the measurement sites in order to ensure that the spectra measured by different instruments correspond to the same object and that the measured spectra of reflectance, emissivity, and microwave bright temperature can be combined.

Optical reflectance refers to the targets’ reflectivity within the wavelength of 350 nm-2500 nm. It was measured by different instruments, including ASD FieldSpec3 Hi-Res, ASD Fieldspec 4 Hi-Res, PSR-3500 and SVC HR1024. The ASD spectroradiometer has a spectral resolution of 3 nm in the VIS-NIR range (350–1000), and 10 nm in the SWIR range (1000–2500 nm) with 2151 channels. The SVC HR1024 has a spectral resolution of approximately 3.5 nm in 350–1000 nm range, 9.5 nm in 1000–1850 nm, and 6.5 nm in 1850–2500 nm with 1024 channels in total. The instrument was set in automatic recording mode, and a measurement was recorded as an average of continuous scans. The number of scans ranged between 5 and 10 per object, and this was automatically set by the instrument. The reflectance spectra were collected at the nadir view angle if not for a specific view angle. For each measurement, the reflectance spectrum of the white reference panel was measured before and after the spectra of the ground object were measured for instrument calibration. Using the whiteboard, the surface reflectance was converted to absolute reflectance. With consideration of the possibility of surface heterogeneity, the spectra measurements were repeated at least three times at different positions or samples for each target. In this way, the spatial representativeness and measuring accuracy were ensured to collect the typical reflectance of one specific target. It is important to point out that the spectra data have been collected by many institutes, and the white reference panel was not the same one during the experimental period. But we have made a unified calibration of these white reference panels before the field experiments in order to make the reflectance measured by different institutes to be consistent. To measure the spectra of forest canopy, a remote sensing vehicle platform (with the maximum height up to 25 m) has been used to measure the top of canopy spectral reflectance.

The land surface emissivity was measured by the Model 102 Portable Field Spectrometer (short for 102 F) from 2–16 μm and ABB BOMEM MR304 Fourier transform infrared spectroscopy with a wavelength range of 2–15 μm. A controlled and portable blackbody has equipped to calibrate the instrument itself and correct the measurement spectral, and a diffuse golden plate was used to collect the atmospheric downward irradiance. To reduce noise, the averaging spectrum from 8 repeated scanning spectra was typically set according to the land surface homogeneity. Because the spectral emissivity depends on the potential temperature of the target, a cooler temperature and a warmer temperature are both required for the blackbody. To measure the infrared emissivity of forest canopy, we have broken off branches from the forests and the component emissivity was measured.

The microwave brightness temperature was measured by three microwave radiometers: JYRS, RPG-1, and RPG-2 at 4 frequencies (1.4 GHz, 6.5 GHz, 10.65 GHz, 18.7 GHz, and 37 GHz) and 2 polarizations (HH and VV). Table 1 lists the detailed technical parameters of the microwave instruments. Generally, the upward microwave radiation was measured by the truck-mounted microwave instrument, and the downward microwave radiation of the Earth surface was measured by a ground-based microwave instrument. For the truck-mounted observation mode, the observation height was about 5–6 m for canopy with a small height. In particular, for the forest canopy with a large height (e.g., larch canopies), the observation height can reach 18 m; the zenith angle of the radiometer was set from −60° to 90° with an interval of 10°; and the azimuth angle was generally set to four different directions for reducing the influence of land surface spatial heterogeneity on the measurement. For ground-based observation mode, microwave radiometers were mounted on 1.2 m high tripods to measure the downward microwave radiation, where the receiving antennas faced upward towards the observed object. The observation azimuth angles at 90° intervals (0°, 90°, 180°, and 270°) and the zenith angle at 10° intervals (40°,50° and 60°) were implemented to reduce the observational error^53,54^.

**Table 1:** Instruments for measuring microwave radiation signals.

Instruments	Frequencies (GHz)	Polarizations	Observation platforms
JYRS	1.4,10.65,18.7 and 36.5	HH and VV	Truck-based/Ground-based
RPG-1	6.5, 10.65, 18.7 and 37	HH and VV	Truck-based
RPG-2	1.4, 6.5 and 10.65	HH and VV	Truck-based

The full-band spectral measurements were conducted over eight land cover types, including four vegetation types (i.e., forest (larch forest), shrub, grass and maize crop), two soil types (i.e., ordinary soil and frozen soil), snow, and cement road (Table 2). Vegetation, soil, and snow were selected because they were the dominant land cover types of the Earth surface, and the spectra of these types show different characteristics over different wavelengths due to the object properties and status differences. The experiments of synchronized measurements were carried out in 2017 over Chengde, Zhangjiakou, and Changchun. The spectra profile for each specific target was estimated as the average of all replicated reflectance profiles. In order to produce a smooth and continuous spectral curve, the least-square polynomial fitting algorithm was applied to remove the abnormal fluctuations in the vapor absorption channels, and the piecewise linear interpolation function was also used to smooth the spectral curve to 1 nm spectral resolution. All the originally measured spectra were processed in generic formats.

**Table 2:** Information of the full-wave-spectrum samples.

No.	Sample	Picture	Locality	Date (YYYYMMDD)	Band wavelength
1	larch		Chengde	20170911	refl_canopy
					emis_canopy
					mwra_canopy
2	shrub		Chengde	20170912	refl_canopy
					emis_canopy
					mwra_canopy
3	grass		Chengde	20170911	refl_canopy
					emis_canopy
					mwra_canopy
4	maize		Zhangjiakou	20170625	refl_canopy
					emis_canopy
					mwra_canopy
5	soil		Chengde	20170911	refl_canopy
					emis_canopy
					mwra_canopy
6	frozen-soil		Changchun	20170110	refl_canopy
					emis_canopy
					mwra_canopy
7	snow		Changchun	20170114	refl_canopy
					emis_canopy
					mwra_canopy
8	cement road		Chengde	20170912	refl_canopy
					emis_canopy
					mwra_canopy

#### Multi-angle reflectance spectra measurements

**Fig. 4:**
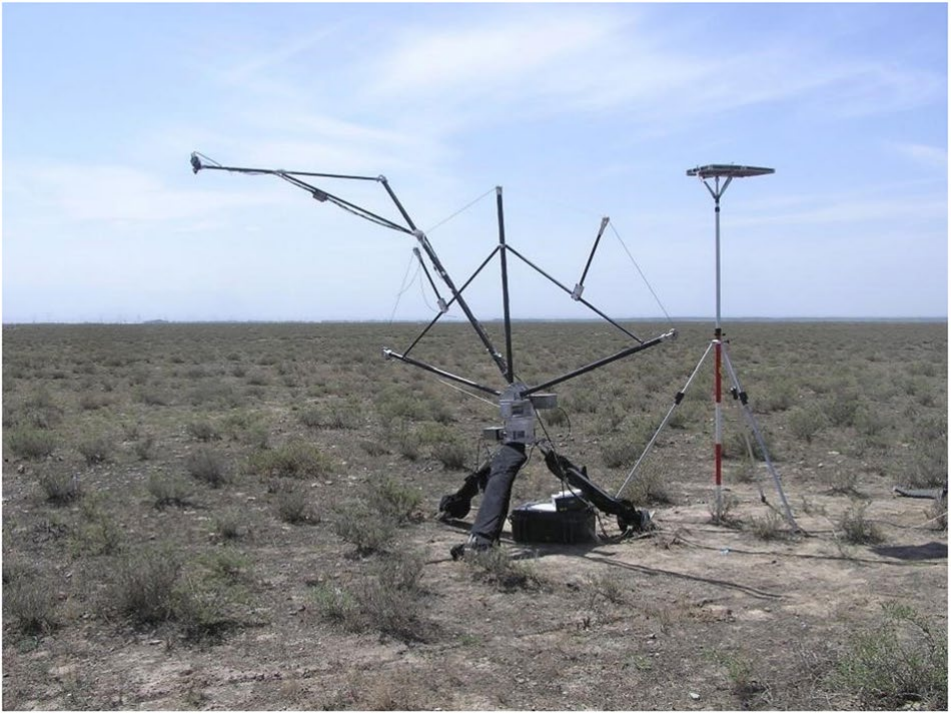
A typical multi-angular reflectance spectra measurement with a portable multi-angle observation device.

Since the BRF is a function of solar and sensor geometry, a multi-angle measurement device was utilized to control the viewing angles when performing the anisotropic reflectance measurements. The typical multi-angle reflectance observation is to change the view angle with a portable multi-angle observation device as shown in Fig. 4. The optic fibre was mounted in the multi-angular device and rotated along the arch to change the view angles with a fixed center at the ground. It is important to note that the field-of-view becomes larger as the view angle increases. Therefore, relatively homogenous areas were selected to suppress the effects of the observed scenes’ variation in different viewing angles. In the measurement of one target, the view zenith angle ranged from −60° to 60° with an interval of 10°. Different observing planes were measured along or across the solar principal plane.

Considering the difficulty in the measurement of all directions of the hemisphere, only a few typical planes were selected for multi-angle observations in the field. The principal plane and the cross-principal plane were selected because the reflectances show the strongest and the weakest changes with different sensor view directions in these two planes. Such multi-angular measurements were implemented over desert vegetation, maize, snow, and soil (chernozems) (Table 3). The first campaign took place in the Gobi covered by moss crust in Zhangye of Gansu Province on Jul 8th, 2012, and 31 planes were nearly continuously measured from morning to afternoon by SVC HR-1024. The number of angles per plane ranged between 9 and 16. In this way, abundant solar illumination angles were collected except for the viewing angular variation. The datasets of maize were collected in Wuxing village of Zhangye on June 29, 2012 with SVC HR-1024. A total of 7 planes were measured with 14 angles per plane. The datasets of snow were measured in Aletai of Xingjiang Province on March 23 and March 24, 2016, with 3 planes and 4 planes, respectively, by ASD, each with 42 angles of measurements. Bare soils with different roughness and moisture in Changchun City of Jilin Province were measured on October 30, 2017, with 12 planes by PSR-3500. For each plane, the number of observation angles ranged between 28 and 29.

**Table 3:** Information of the multi-angular reflectance samples.

NO.	Samples	Locality	Starting time	Ending time	View planes
1	Desert Vegetion	Zhangye	20120708112630	20120708173000	31
2	Maize	Zhangye	20120629124100	20120629172200	7
3	Chernozems	Changchun	20171030123000	20171030144700	12
4	Snow	Aletai	20160323122001	20160324161101	7

#### Multi-scale reflectance spectra measurements

The multiscale reflectance spectra were measured considering the effects of vegetation structure, spatial heterogeneity, and spectral resolution on reflectance. To investigate the scaling dependency of the measured spectra, both components (leaf) and canopy spectra of typical forest and crops were measured at the ground or near the ground by portable setup or tower-borne platforms, respectively. Moreover, the 30 m Landsat ETM/OLI and 500 m MODIS pixel surface reflectances were also extracted to match the ground/near-ground measurements at the nearest time when the ground or near-ground measurements were carried out. It is important to point out that the inclusion of Landsat ETM/OLI and MODIS surface reflectances is not for direct comparison between the spectra data with different spatial scales, but serves as a complement to the measured spectra on the leaf and canopy scales, which is important to understand the variation of spectra characteristics on different spatial scales more intuitively. The reflectance spectra were collected at the nadir view angle if not for a specific view angle.

The measurements were carried out over the main forests and crops of different areas in China (Supplementary Table 1). The croplands’ spectra were sampled from homogenous large-area-covered quadrates. For the forest canopy, the field measurement was assisted by a tower or UVA. ASD or SVC was used to measure the spectra at the ground or from the tower. While for UVA, the hyperspectral imager was mounted to measure canopy spectra. The multiscale measurements were first carried out in 2004, and the majority of measurements were conducted in the growing season (from July to October) of 2017 and 2018. The MODIS and Landsat ETM/OLI pixel land surface reflectance corresponding to *in situ* measurements were also extracted to form the multiscale reflectance spectra from leaf to canopy and then to ecosystem.

#### Temporal dynamic field spectral measurements

The temporal dynamic field spectral measurements were carried out for vegetation-covered surfaces in the growing season in order to understand the relationship between reflectance spectra and the growing status under the clear sky conditions. Only optical reflectance was measured by ASD Fieldspec3 in the range between 325 nm and 900 nm in 2013, while the others were measured by Fieldspec4 Hi-Res ranging from 350 nm to 2500 nm. The reflectance spectra were also collected at the nadir view angle if not specified.

The continuous reflectance spectra measurement was implemented within the growing season in 4 typical agriculture test sites of China: Changchun, Zhangye, and Haibei Tibetan Autonomous Prefecture. The measurements in Changchun were carried out in 2016. Several typical vegetation types such as maize, rice, wheat, potato, soybean, and mungbean were included (Table 4). The number of sampling days ranged from 10 to 16, covering the whole growing season. The vegetation temporal dynamic reflectance spectra in Zhangye were collected in 2013. The measured vegetation types included rape, barely, corn, and onion, and the number of sampling days ranged between 9 and 12. For each vegetation type, there were as least two reflectance spectra measurements for each month during the experimental period. In this way, the measured reflectance spectra of this vegetation nearly represent the temporal dynamics in the growing season. The measurements in Haibei Tibetan Autonomous Prefecture were also conducted in 2013. Only the reflectance spectra of grassland were collected from May to September in 13 sampling days.

**Table 4:** Information of the temporal dynamics of vegetation reflectance samples.

NO.	Samples	Locality	Starting date	Ending date	Sampling days
1	maize	Changchun	20160419	20161026	16
2	rice	Changchun	20160419	20161026	12
3	wheat	Changchun	20160419	20161026	15
4	potato	Changchun	20160510	20161026	14
5	soybean	Changchun	20160419	20161026	14
6	mungbean	Changchun	20160527	20161026	10
7	rape	Zhangye	20130603	20130927	10
8	barley	Zhangye	20130612	20130927	9
9	corn	Zhangye	20130511	20130928	12
10	onion	Zhangye	20130511	20130728	9
11	grassland	Haibei Tibetan Autonomous Prefecture	20130524	20130927	13

## Data Records

The datasets are available at the National Tibetan Plateau Data Center (https://data.tpdc.ac.cn/home). The datasets here include the full-band spectra dataset (10.11888/RemoteSen.tpdc.272930, https://cstr.cn/18406.11.RemoteSen.tpdc.272930)^55^, multi-angle spectra dataset (10.11888/RemoteSen.tpdc.272931, https://cstr.cn/18406.11.RemoteSen.tpdc.272931)^56^, multi-scale spectra dataset (10.11888/RemoteSen.tpdc.272932, https://cstr.cn/18406.11.RemoteSen.tpdc.272932)^57^, and temporal dynamic spectra dataset (10.11888/RemoteSen.tpdc.272929, https://cstr.cn/18406.11.RemoteSen.tpdc.272929)^58^. The data in these datsets have passed rigorous quality assurance tests.

### Full-band spectra dataset

**Fig. 5:**
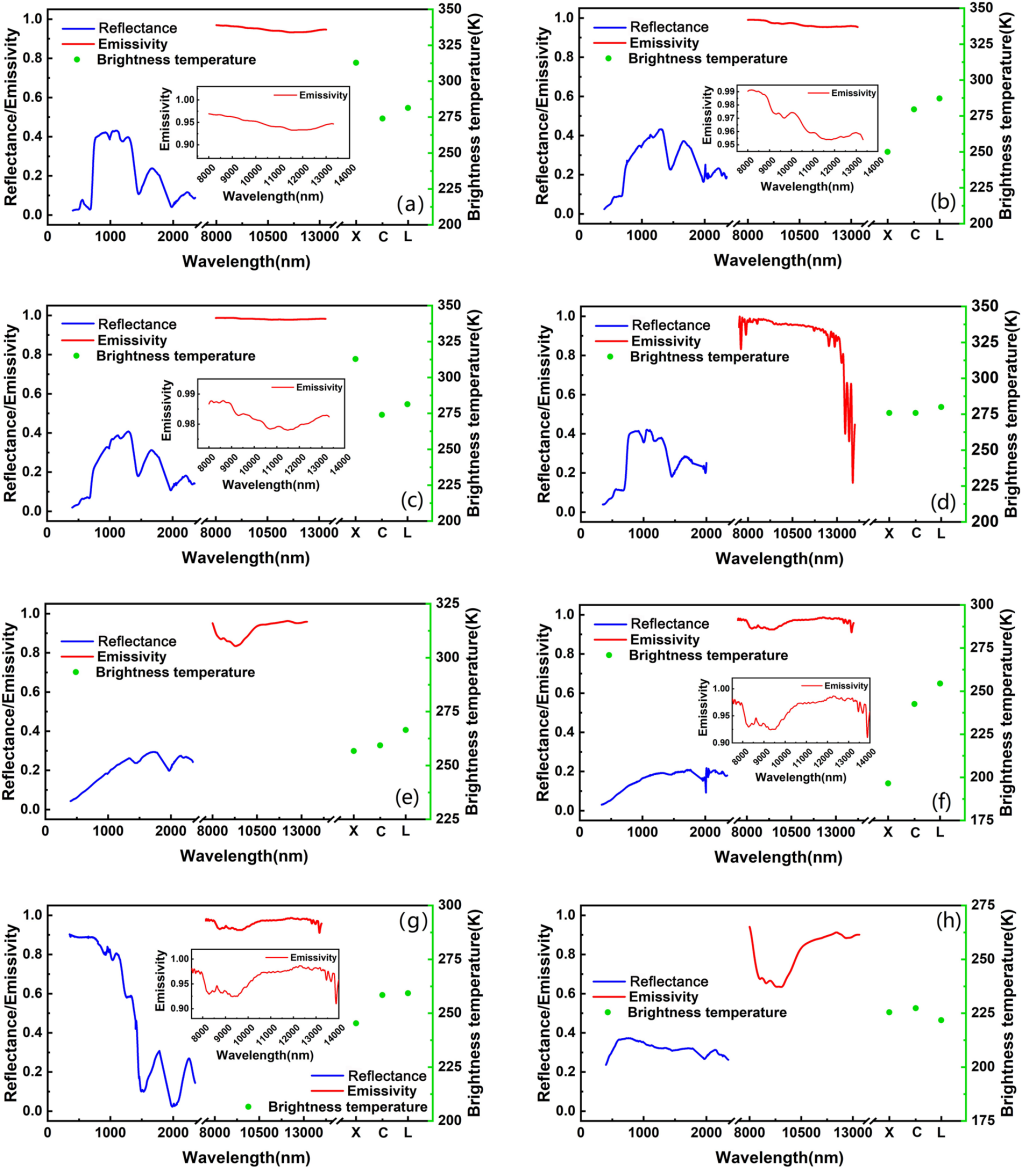
The full-spectrum of typical land cover types. (**a**) larch, (**b**) shrub, (**c**) grass, (**d**) maize, (**e**) normal soil, (**f**) frozen soil, (**g**) cement road, and (**h**) snow.

Figure 5 shows the typic full-band spectra of grass, larch, maize, shrub, cement, snow, frozen soil, and normal soil. Here, the reflectance, emissivity and microwave radiation are of nadir view while the Sun illumination angle was calculated according to the observation time and location. As shown in Fig. 5a–d, the reflectance spectra over vegetation cover tends to polarize into two groups: larch and maize show similar reflectance spectra while shrub and grass display similar reflectance spectra. The former presents the typical spectral characteristics of vegetation while the latter does not. This occurs because the land surface is almost entirely covered by larch and maize as shown in the picture in Table 2. By contrast, the surface covered by shrub and grass is actually a mixture of withered plants and soil as shown in the picture in Table 2. Nevertheless, although larch and maize display similar reflectance spectra in the VNIR-SWIR spectral domain, they present large differences in the emissivity in the TIR spectral domain and microwave spectral domains (Fig. 5a,d). Larch has a relatively flat signal from 8 µm to 13.5 µm. By contrast, maize presents a rapidly decreasing signal around 13 µm. A reverse phenomenon appears in the microwave spectral domains where larch shows a relatively large difference in signal in the X, C, and L bands while maize shows relatively stable signal in these bands. Similar results can be found for shrub and grass (Fig. 5b,c), where a relatively large difference in signal appears in the TIR spectral domain and microwave spectral domain, despite their similarity in the VNIR-SWIR spectral domain. As shown in Fig. 5e,f, the spectra of soil and frozen soil not only show different magnitudes of reflectance and emissivity but also present different characteristics with the wavelength. These results demonstrate that the full-spectrum spectra provide more useful information for characterizing the spectral endmembers of remote sensing models, thus support more accurate identification and classification of surface type.

Associated with the spectra, the parameters describing the targets’ properties such as the object type, observed time, location, the solar and sensor geometry, satellite images as well as the observed instrument and contributions of units, were simultaneously recorded as auxiliary information to describe the spectral measurement scene (Table 5). The satellite pixel spectra (i.e., 30 m Landsat ETM/OLI surface reflectance and 500 m MODIS surface reflectance) corresponding to the measured site were also extracted. The satellite spectra and the field spectra can be matched by convolving the field spectra with the satellite’s sensor sampling and band channel. This combination of ground-based and satellite-derived spectra can reflect the object spectral characteristics at different spatial scales. The 30 m Landsat ETM/OLI surface reflectance and 500-m MODIS surface reflectance as well as their metadata were collected and processed as the standard files.

**Table 5:** The auxiliary parameters for the spectra dataset in the metadata file.

No.	Category	Items
1	Object type	Object name, land cover
2	Observed location and time	Location name, longitude, latitude, measurement time
3	Weather condition	Sunlight, cloud amount, visibility
4	Instrument formation	Name, spectral range and resolution, field of view
5	Measurement information	Platform, observation height, number of measurements
6	Solar and sensor geometry	Solar zenith, solar azimuth, sensor zenith, sensor azimuth
7	Site and its around view	Photos of object and satellite quick view image.
8	Contributions unit	Unit, author name, contacted information

The full-band spectra dataset includes 8 full-spectrum data records. Each record was organized in a folder named “AAAA_BBBB_CCCC_YYYYMMDDHHMMSS_XXXX”. “AAAA” and “XXXX” were set as “spec” and “B001”, respectively. “BBBB_CCCC” represents land cover and object name. “YYYYMMDDHHMMSS” shows the measured time. One example of the folder name is “spec_canopy_maize_20160625100500_B001”. The core files of the full-spectrum include the combination data of reflectance, emissivity, microwave bright temperature, metadata files, and satellite data. Each data file was processed as TXT format and the metadata file was set as XML format. The satellite data and the quick view image were set as Tiff and JPEG format, respectively. The file name is recorded as “AAAA_BBBB_CCCC_YYYYMMDDHHMMSS_XXXX”. The naming rule of the file is summarized in Table 6.

**Table 6:** The meaning of the fields in the name of the spectra data.

Fields	Meaning	Value set
AAAA	Spectral type	“refl”, “emis”, and “mwra” denote reflectance, emissivity, and microwave radiation, respectively;“pixel”, “img”, and “view” represent the satellite pixel spectral, the satellite image, and the quick-view image, respectively; “comp” refer to the general information of the spectra metadata; “pixel” indicates the information of satellite pixel spectral metadata.
BBBB	Land cover	Canopy, snow, soil for examples
CCCC	Object name	Grass, shrubbery, maize, frozen soil for examples
YYYY	Year	2017, 2016, for examples
MMDD	Month and Day	Taking “0625” for an example, June 25th
HHMMSS	Time	Taking “100500” for an example, 10:05 am
XXXX	File type	“A001” means the data file, and “B001” means the auxiliary file of XML.

### Multi-angular reflectance spectra dataset

**Fig. 6:**
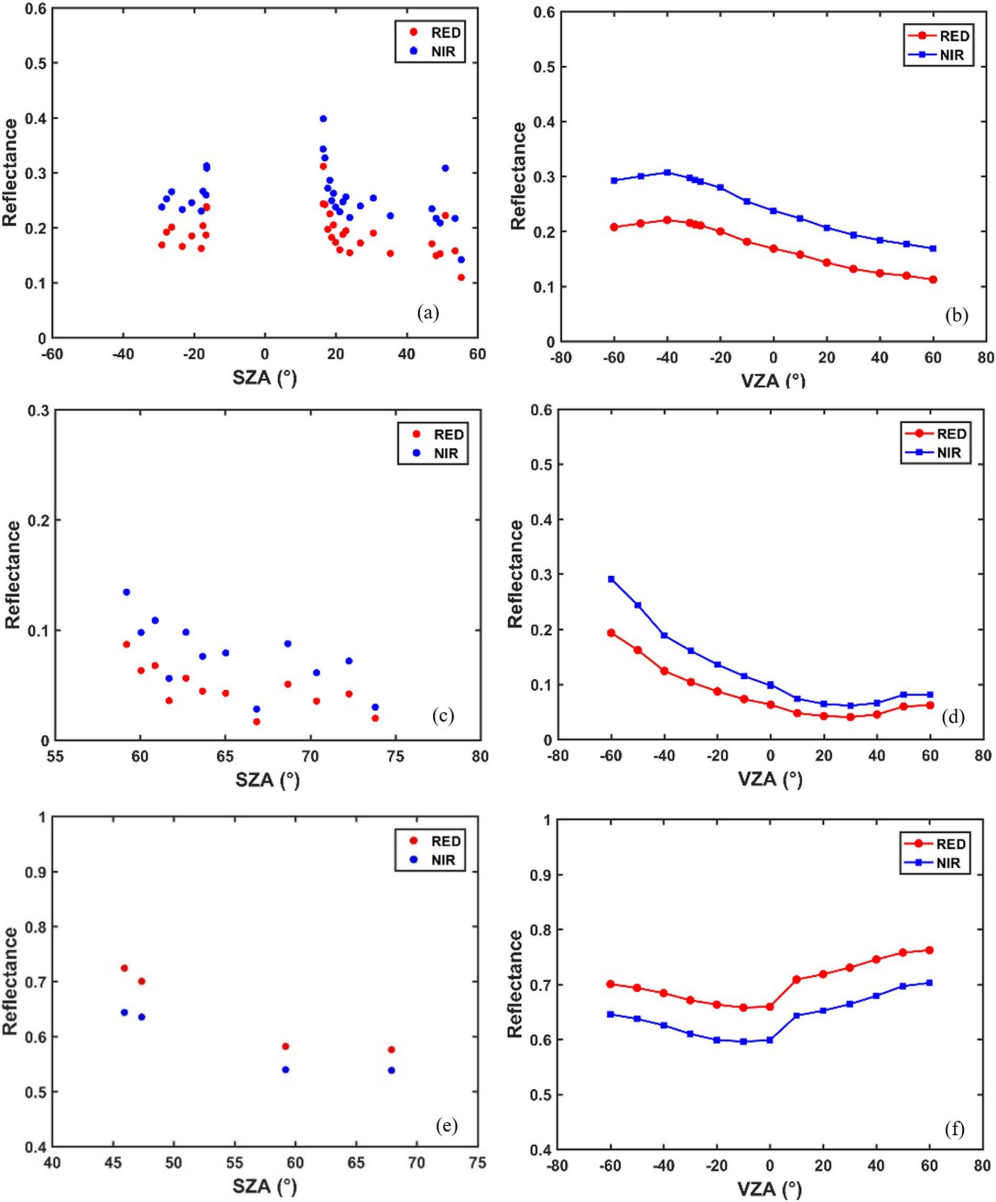
The reflectance variation with SZA and VZA of desert vegetation (**a,b**), soil (**c,d**) and snow (**e,f**).

Figure 6 shows the variation of reflectance with the solar zenith angles (SZA) and view angles (VZA) over the three typical land surfaces (i.e., desert vegetation, soil, and snow). It can be seen that all of them show a significant variation with SZA. Especially, as shown in Fig. 6a,c,e, the reflectance decreased gradually as the solar zenith angle increased. When the solar zenith angle was fixed, the reflectance also changed with the sensor zenith angle. Desert vegetation shows a hot spot indicated by the largest reflectance. However, snow and soil did not show the hot spot due to the large solar zenith angle, as it is very difficult to observe the same angle as the Sun in the measurement process.

The multi-angular measurements in each plane were organized in a folder named “AAAA_BBBB_CCCC_YYYYMMDDHHMMSS”, and there are 57 folders in total. The “AAAA” was set as “BRDF”. The “BBBB_CCCC” and “YYYYMMDDHHMMSS” imply the same as those in Table 6. The spectra data in different angles and their metadata file were also processed as TXT format and XML format, respectively. The file of the measured reflectance spectra in a specific angle was named as “AAAA_BBBB_CCCC_YYYYMMDDHHMMSS_XXXX”. The naming scheme of “AAAA_BBBB_CCCC_YYYYMMDDHHMMSS” is the same as the folder. While the “XXXX” present the measurement angles. For instance, “A001” and “A015” refer to the measurement in the first and the fifteenth angles, respectively. The “AAAA” of the metadata files were set as “comp” and “chrp”. The former saves the general information of the multiangle measurements while the latter record the special scene information of the spectra. Since the collected fine-scale and coarse-scale satellite reflectance were only with one observed angle, they were not included in each folder.

### Multi-scale vegetation reflectance spectra dataset

**Fig. 7:**
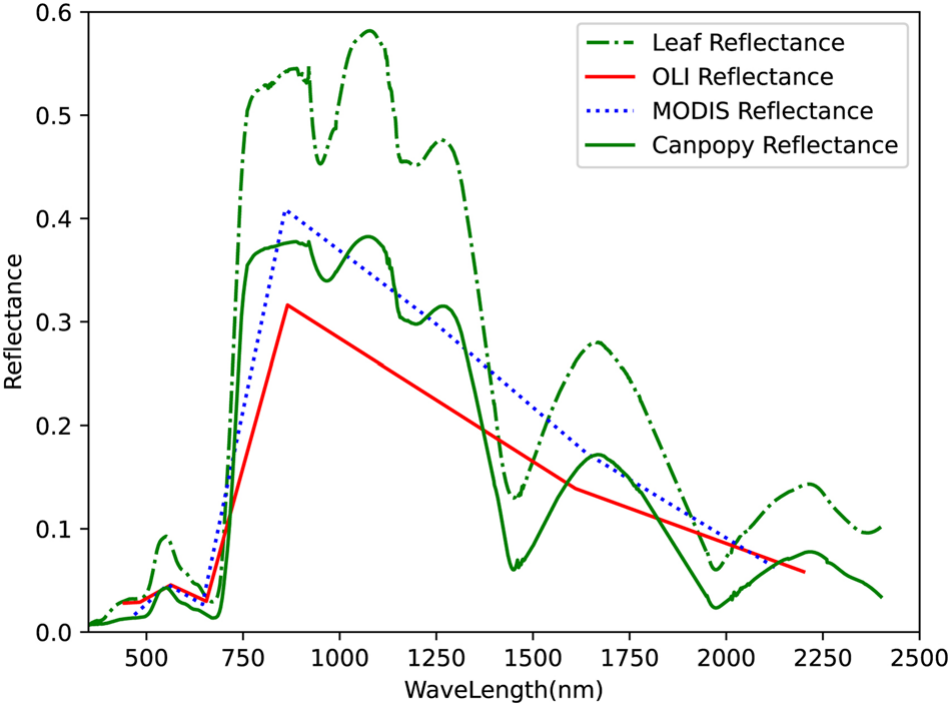
Multiscale vegetation spectra from leaf and canopy to satellite scales.

Figure 7 presents the surface reflectance spectra measured at different spatial scales. The comparison at different spatial scales partly aims to understand how the coarse-resolution pixel reflectance spectra are composed for different types of heterogeneous land surfaces, and partly supports the development of validation methods with consideration of the surface heterogeneity and spatial scale differences. In Fig. 7, there are obvious differences in vegetation reflectance at different spatial scales. But there is a strong relationship between leaf and canopy reflectance. This relationship has been confirmed by many remote sensing radiation transfer models such as PROSECT and PROSAIL. Hence, this multiscale reflectance spectra dataset can be considered representative and has the advantage of being coincident in time and space. The results also indicate that leaf, canopy as well as reflectance measured by satellite at different scales are necessary to characterize their object states and radiation regime at different spatial scales.

The leaf-canopy-Landsat-MODIS spectra data matched in time and space were organized in a folder named “AAAA_BBBB_CCCC_YYYYMMDDHHMMSS”, and there are 31 folders in total. The “AAAA” was set as “spec”. The “BBBB_CCCC” and “YYYYMMDDHHMMSS” imply the same as those in Table 6. The core files of the multiscale spectra data include the combination data of leaf and canopy reflectance, metadata files, and satellite data. The formats of the data file, metadata file, satellite data, and quick view images were the same as those of full-spectrum spectra dataset. The naming schemes of these files were also the same as Table 6. For leaf and canopy reflectance spectra, “BBBB” were set as “compo” and “canopy”, respectively.

### Temporal dynamic vegetation reflectance spectra dataset

**Fig. 8:**
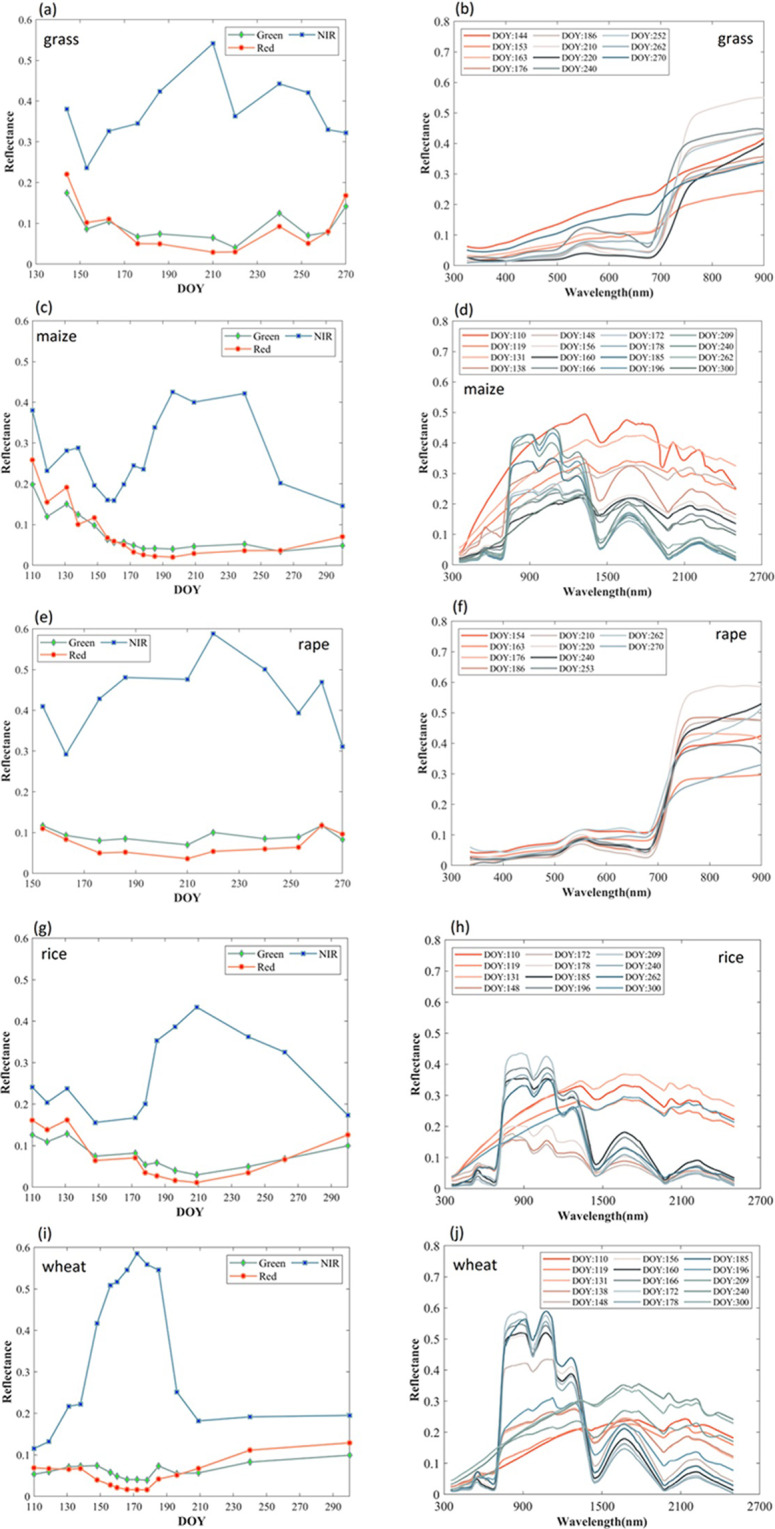
The reflectance variation in different growth stages for grass (**a,b**), maize (**c,d**), rape (**e,f**), rice (**g,h**), and wheat (**I,j**), respectively.

Figure 8 shows the reflectance curve within the growth season at red, green and near-infrared (NIR) bands over some vegetation types. For each vegetation type, the spectra of NIR indicate a gradually increasing trend from bare soil-dominated scenes to full vegetation cover scenes. The reflectance of the canopy decreases again, which shows the vegetation has entered a senescence period. The spectral variability from a typical soil to vegetation shows the canopy cover at different stages of the vegetation growing season.

For each vegetation type, the reflectance spectra measurements for each sampling day were organized in a folder named “AAAA_BBBB_CCCC_YYYYMMDDHHMMSS”. There were 134 folders in total. The naming of these folders follows the same convention as for the full-spectrum spectra dataset. The core files in each folder, their format, and the naming scheme of these files were the same as those of the full-band spectra dataset. For each specific vegetation type, all these reflectance measurements during the vegetation growth period were grouped by the measuring time to form the vegetation temporal dynamics reflectance spectra data.

## Technical Validation

### Error and uncertainty

The field spectra measurements were affected by many factors leading to errors and uncertainties, which complicate the understanding of the interaction between the measured target and the electromagnetic radiation. These factors can be grouped into three types: instrumental characteristics, prevailing conditions, and measuring operation and data processing.

For the instruments, device configuration precisions such as the radiometric response, wavelength accuracy, and signal to noise ratio were collected from their company. However, instruments’ degradation and drift are unavoidable. For instance, the FOVs of the ASD spectroradiometer are different from the nominal FOV reported by the manufacturer and the sensor responsivity within the FOV is nonuniform^59^. Another example is the white reference panel which was necessary for field measurements. It is generally assumed to be a Lambertian surface. However, its reflectance may show an anisotropic behavior, depending on the illumination and view geometry^60^. This results in system uncertainty in the absolute reflectance values. Furthermore, the reference panel suffers from degradation. To minimize such effects, radiometric and wavelength calibration of the instruments and reference panel was regularly calibrated in the laboratory. However, it should be pointed out that the calibration is never perfect^61^. Except for the instruments, assisting devices also bring in errors. For example, the angle control precision of the multi-angular system was about 2°. In fact, the characteristics of the instruments are difficult to determine. Finally, it is important to note that these spectra data were collected by different instruments and by different institutes, hence, errors and inconsistency may be introduced. The complexity of these factors in the field spectra measurements highlights the importance of the comprehensive metadata of the field measurement to make better use of this dataset. Additionally, to reduce such uncertainties, every effort has been made possible to minimize errors and uncertainties (e.g., sampling, measuring, and processing procedure).

During measurements and in between, instability from the environmental conditions (e.g., varying illumination and atmospheric composition) and the target status will bring some uncertainties to the measurements. Additionally, the possible obstructive or reflective objects in the surroundings of the measurement also introduce uncertainty to the measured spectra. Compared to the reflectance measurements, environmental and target status bring more uncertainties for the measurements in thermal emission and microwave spectra due to the longer duration required for an accurate reading. To reduce such effects, campaigns were conducted under clear sky with relatively stable weather conditions, and the measuring of the thermal and microwave spectra was carried out in an open space to avoid the surrounding radiation disturbances. The wind force was required to be less than Level 3, and the horizontal visibility was required to be larger than 10 km.

It is important to point out that the uncorrected irradiance levels together with other external sources may compensate for each other, and the resulting spectra would not suffer so much uncertainty. To further reduce the random errors caused by these factors, the field measurements had been repeated several times of the same target, and the average is used in the final spectra dataset. Averaging reduces data variations due to the instability influences to a certain degree.

### Applications

As field-measured spectra were collected from homogenous large-area-covered areas with abundant spatial/temporal or even angular variations, they are representative in temporal and spatial dimensions thus feasible for modeling, inversion, and validation in quantitative analyses of remote sensing data. The full-band spectra characterized the same target in different waveband ranges, it will be undoubtedly helpful to investigate the radiative transfer mechanism among different waveband ranges, which may provide a solution to combine the information of the optical, infrared, and microwave observations to retrieve the land surface parameters. The radiation regime of the main wavelength supports the research of quantitative remote sensing radiative transfer models, the surface variable inversion of the Earth system, validation of remote sensing products, more detailed classification of ground cover, and so on.

Anisotropic reflectance is the basic property of an object that characterizes the hemispherically unequal scattering radiation. Signals received by a satellite sensor always vary with the varying illumination-observation geometries, which are common in remote sensing images. The multi-angular measurements are desirable for the anisotropic modelling and the related parameters retrieval and validation. The reflectance spectra have been utilized to validate the MODIS NDVI product^62^ or the BRDF modeling assessment^12^. Besides, these data are also helpful for validation of model and/or algorithm development. For example, You *et al*.^63^ and Wen *et al*.^64^ have compared the retrieval BRDF shapes derived from models with the *in-situ* angular reflectance data.

In addition to the illumination-observation geometry, vegetation spectra are dependent on vegetation structure and spatial heterogeneity. Taking one canopy as an example, its reflectance is collectively contributed by the leaf reflectance and the vegetation structure, and the measurements at different spatial scales will reveal very different reflectance signals. Our multiscale vegetation reflectance spectra dataset, including the leaf, canopy, and satellite remote sensing reflectance at different spatial scales, are necessary to show their object states and radiation regimes at different scales. It has great potential in environment and agricultural monitoring, the study of climate change, the research on spatial scale effects, and the validation of remote sensing models and satellite products. For instance, the component spectra of soil and leaves have been utilized in a BRDF model parameterization as prior-knowledge to simplify the model for easy-and-fast retrieval by You *et al*.^13^.

**Fig. 9:**
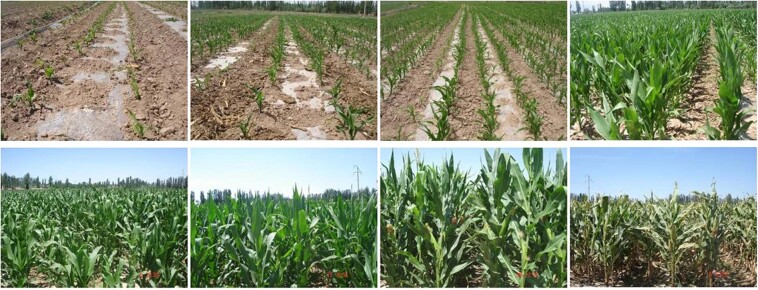
Canopy cover at different growth stages of a corn field.

The spectra of vegetation at different growth stages show significantly different shapes due to changes in structural and physiochemical properties. Taking corn field as an example (Fig. 9), vegetation cover changes with the growth from a soil-similar scene to a vegetation-similar scene. As a result, the surface reflectance spectra change from that of bare soil to that of vegetation as canopy develops. Hence, the temporal dynamic spectra of typical vegetation are crucial to understand the relationship between vegetation growth status and the reflectance spectra. This temporal dynamic reflectance spectra dataset is crucial for analyzing vegetation dynamics and good for vegetation growth modeling and research on land surface phenology or vegetation green-up.

In general, the presented datasets are promising for further research in remote sensing modeling and inversion. Various types of soil, vegetation, and snow reflectance support the modelling development, such as the parameterization of key variables in the BRDF model^12^ and the training of the narrowband albedo to broadband albedo conversion coefficients^11^. Moreover, they also play a crucial role in monitoring global vegetation dynamics, surface energy budget, and land surface processes, and can serve as direct validation reference information for satellite measurements.

### Limitations of the dataset

Although the error and uncertainty of the field-measured spectra have been analyzed, the effect of different factors has not been quantified or corrected in our measurements. It is also difficult to quantify uncertainty of field measurements taken under natural conditions, as the exact same conditions cannot be replicated. In addition, there are limiting factors in the selection of measurement samples. Although a homogeneous area was selected to ensure the spatial representativeness of field measurements by different instruments, the sample homogeneity was merely visually determined. Moreover, the samples measured do not cover each and every land surface type in China. They were selected following two principles: the feasibility and convenience of field measurements; the representativeness of the land surface. The areas where there are field stations were selected first, and the typical surface types around the field stations were identified. Then the relatively homogeneous areas were selected for each typical surface type to conduct the field spectra measurements. The quantitative evaluation of the spectral shape was not conducted and the comparison with the reflectance spectral library such as USGS was not made. Nevertheless, the influence of these limitations on the usage of datasets can be decreased, as we have provided comprehensive metadata of the field measurements for good utilization.

## Supplementary information

Supplementary Table 1

## Data Availability

The spectra data were processed and analyzed using Matlab 2016a. All used scripts to implement are available at Github: https://github.com/hanyyuan/groundObjectSpectrum.git.

## References

[1] Asadzadeh S, de Souza Filho CR (2016). A review on spectral processing methods for geological remote sensing. International Journal of Applied Earth Observation and Geoinformation.

[2] Koirala, B., Zahiri, Z., Lamberti, A., & Scheunders, P. Robust supervised method for nonlinear spectral unmixing accounting for endmember variability. *IEEE Transactions on Geoscience and Remote Sensing*, 59-9, 10.1109/TGRS.2020.3031012 (2021).

[3] Powell RL, Roberts DA, Dennison PE, Hess LL (2007). Sub-pixel mapping of urban land cover using multiple endmember spectral mixture analysis: Manaus, Brazil. Remote Sensing of Environment.

[4] Metsämäki S (2012). An optical reflectance model-based method for fractional snow cover mapping applicable to continental scale. Remote Sensing of Environment.

[5] Hansen MC, Loveland TR (2012). A review of large area monitoring of land cover change using Landsat data. Remote Sensing of Environment.

[6] Zhang X, Liu LY, Chen XD, Xie S, Gao Y (2019). Fine land-cover mapping in China using landsat datacube and an operational speclib-based approach. Remote Sensing.

[7] Herold M, Roberts DA, Gardner ME, Dennison PE (2004). Spectrometry for urban area remote sensing—Development and analysis of a spectral library from 350 to 2400 nm. Remote Sensing of Environment.

[8] Jin C, Shen MG, Zhu XL, Tang YH (2009). Indicator of flower status derived from *in situ* hyperspectral measurement in an alpine meadow on the Tibetan Plateau. Ecological Indicators.

[9] Lammoglia T, de Souza Filho CR (2011). Spectroscopic characterization of oils yielded from Brazilian offshore basins: Potential applications of remote sensing. Remote Sensing of Environment.

[10] Gerhards M, Schler FM, Mallick K, Udelhoven T (2019). Challenges and Future Perspectives of Multi-/Hyperspectral Thermal Infrared Remote Sensing for Crop Water-Stress Detection: A Review. Remote Sensing.

[11] Peng S (2017). Multi-Staged NDVI Dependent Snow-Free Land-Surface Shortwave Albedo Narrowband-to-Broadband (NTB) Coefficients and Their Sensitivity Analysis. Remote Sensing.

[12] Wu SB (2019). Derivation of Kernel Functions for Kernel-Driven Reflectance Model Over Sloping Terrain. IEEE Journal of Selected Topics in Applied Earth Observations and Remote Sensing.

[13] You D (2020). The Component-Spectra-Parameterized Angular and Spectral Kernel-Driven Model: A Potential Solution for Global BRDF/Albedo Retrieval From Multisensor Satellite Data. IEEE Transactions on Geoscience and Remote Sensing.

[14] Bartlett DS, Klemas V (1981). *In situ* spectral reflectance studies of tidal wetland grasses. Photogrammetric Engineering and Remote Sensing.

[15] Frouin R, Schwindling M, Deschamps PY (1996). Spectral reflectance of sea foam in the visible and near‐infrared: *In situ* measurements and remote sensing implications. Journal of Geophysical Research: Oceans.

[16] Das DK (2013). Spectral reflectance characteristics of healthy and yellow mosaic virus infected soybean (Glycine max L.) leaves in a semiarid environment. Journal of Agrometeorology.

[17] Tanikawa T (2014). *In-situ* measurement of polarization properties of snow surface under the Brewster geometry in Hokkaido, Japan and northwest Greenland ice sheet. Journal of Geophysical Research: Atmospheres.

[18] Pirazzini R, Räisänen P, Vihma T, Johansson M, Tastula E-M (2015). Measurements and modelling of snow particle size and shortwave infrared albedo over a melting Antarctic ice sheet. The Cryosphere.

[19] Ma S (2019). Application of the water-related spectral reflectance indices: A review. Ecological indicators.

[20] Hideki K (2018). *In situ* observations reveal how spectral reflectance responds to growing season phenology of an open evergreen forest in Alaska. Remote Sensing.

[21] Prudnikova E, Savin I, Vindeker G, Grubina P, Sharychev D (2019). Influence of soil background on spectral reflectance of winter wheat crop canopy. Remote Sensing.

[22] Cheng J (2020). Review of Methods and Remote Sensing Cases Using Spectral Library. Remote Sensing Technology and Application.

[23] Clark, R. N., Swayze, G. A., Wise, R., Livo, E. & Sutley, S. J. USGS digital spectral library splib06a. *US Geological Survey* **231**, 10.3133/ds231 (2007).

[24] Kokaly, R. F., Clark, R. N., Swayze, G. A., Livo, K. E. & Hoefen, T. M. USGS Spectral Library Version **7**, 10.3133/ds1035 (2017).

[25] Baldridge AM, Hook SJ, Grove CI, Rivera G (2009). The ASTER spectral library version 2.0. Remote Sensing of Environment.

[26] Christensen PR (2000). A thermal emission spectral library of rock-forming minerals. Journal of Geophysical Research Atmospheres.

[27] Hosgood, B., *et al*. Leaf optical properties experiment 93 (LOPEX93), Report EUR 16095 (1995).

[28] Garrity, D. & Bindraban, P. A globally distributed soil spectral library visible near infrared diffuse reflectance spectra, ICRAF (World Agroforestry Centre)/ISRIC (World Soil Information) Spectral Library: Nairobi, Kenya (2004).

[29] Rossel,Viscarra RA, Behrens B, Brown D, Dematte A, Shepherd D (2016). A global spectral library to characterize the world’s soil. Earth-Science Reviews.

[30] Roberts DA, Quattrochi DA, Hulley GC, Hook SJ, Green R (2012). Synergies between VSWIR and TIR data for the urban environment: An evaluation of the potential for the Hyperspectral Infrared Imager (HyspIRI) Decadal Survey mission. Remote Sensing of Environment.

[31] Kotthaus S, Smith T, Wooster M, Grimmond SB (2014). Derivation of an urban materials spectral library through emittance and reflectance spectroscopy. ISPRS Journal of Photogrammetry and Remote Sensing.

[32] Maturilli A, Helbert J, Ferrari S, Davidsson B, D’Amore M (2016). Characterization of asteroid analogues by means of emission and reflectance spectroscopy in the 1-to 100-µm spectral range. Earth, Planets and Space.

[33] Wen J (2018). Characterizing land surface anisotropic reflectance over rugged terrain: A review of concepts and recent developments. Remote Sensing.

[34] Comar A (2014). ACT: A leaf BRDF model taking into account the azimuthal anisotropy of monocotyledonous leaf surface. Remote Sensing of Environment.

[35] Jiao ZH (2019). Modeling of land surface thermal anisotropy based on directional and equivalent brightness temperatures over complex terrain. IEEE Journal of Selected Topics in Applied Earth Observations and Remote Sensing.

[36] Ren H (2014). Angular normalization of land surface temperature and emissivity using multiangular middle and thermal infrared data. IEEE Transactions on Geoscience and Remote Sensing.

[37] Zhu X, Jin K, Hui Q (2020). Near-field power-focused directional radiation in microwave wireless power transfer system. IEEE Journal of Emerging and Selected Topics in Power Electronics.

[38] Weyermann J, Damm A, Kneubuhler M, Schaepman ME (2013). Correction of reflectance anisotropy effects of vegetation on airborne spectroscopy data and derived products. IEEE Transactions on Geoscience and Remote Sensing.

[39] Hu T, Li H, Cao B, Dijk A, Liu Q (2019). Influence of emissivity angular variation on land surface temperature retrieved using the generalized split-window algorithm. International journal of applied earth observation and geoinformation.

[40] Guillevic PC, Bork-Unkelbach A, Gottsche FM, Hulley G, Gastellu-Etchegorry JP (2013). Directional viewing effects on satellite land surface temperature products over sparse vegetation canopies—A multisensor analysis. IEEE Geoscience and Remote Sensing Letters.

[41] Schlerf M, Atzberger C (2012). Vegetation structure retrieval in beech and spruce forests using spectrodirectional satellite data. IEEE Journal of Selected Topics in Applied Earth Observations and Remote Sensing.

[42] Funk JL (2017). Revisiting the H oly G rail: using plant functional traits to understand ecological processes. Biological Reviews.

[43] Faucon MP, Houben D, Lambers H (2017). Plant functional traits: soil and ecosystem services. Trends in plant science.

[44] Kidane Y, Stahlmann R, Beierkuhnlein C (2012). Vegetation dynamics, and land use and land cover change in the Bale Mountains, Ethiopia. Environmental monitoring and assessment.

[45] Matthews HD, Weaver AJ, Meissner KJ, Gillett NP, Eby M (2004). Natural and anthropogenic climate change: incorporating historical land cover change, vegetation dynamics and the global carbon cycle. Climate Dynamics.

[46] Horion S, Cornet Y, Erpicum M, Tychon B (2013). Studying interactions between climate variability and vegetation dynamic using a phenology based approach. International Journal of Applied Earth Observation and Geoinformation.

[47] Wen JG (2022). Characterizing the Effect of Spatial Heterogeneity and the Deployment of Sampled Plots on the Uncertainty of Ground “Truth” on a Coarse Grid Scale: Case Study for Near-Infrared (NIR) Surface Reflectance. Journal of Geophysical Research: Atmospheres.

[48] Snyder WC, Wan Z, Zhang Y, Feng YZ (1997). Thermal infrared (3–14 μm) bidirectional reflectance measurements of sands and soils. Remote Sensing of Environment.

[49] Hook SJ, Kahle AB (1996). The micro fourier transform interferometer-a new field spectrometer for acquisition of infrared data of natural surface. Remote Sensing of Environment.

[50] Wang N, Wu H, Nerry F, Li C, Li ZL (2011). Temperature and emissivity retrievals from hyperspectral thermal infrared data using linear spectral emissivity constraint. IEEE Transactions on Geoscience and Remote Sensing.

[51] Guo P, Zhao T, Shi J, Xu H, Niu S (2021). Assessing the active-passive approach at variant incidence angles for microwave brightness temperature downscaling. International Journal of Digital Earth.

[52] Mattioli V, Milani L, Magde KM, Brost GA, Marzano FS (2016). Retrieval of sun brightness temperature and precipitating cloud extinction using ground-based sun-tracking microwave radiometry. IEEE Journal of Selected Topics in Applied Earth Observations and Remote Sensing.

[53] Zheng XM, Li XF, Jin MJ, Jiang T, Zhao K (2017). Characteristics of L-band transmissivity and effective scattering albedo of boreal forests: a case study in northeast China. Remote Sensing Letters.

[54] Wang GR (2021). An investigation on microwave transmissivity at frequencies of 18.7 and 36.5 GHz for diverse forest types during snow season. International Journal of Digital Earth.

[55] Wen J (2022). National Tibetan Plateau Data Center.

[56] Wen J (2022). National Tibetan Plateau Data Center.

[57] Wen J (2022). National Tibetan Plateau Data Center.

[58] Wen J (2022). National Tibetan Plateau Data Center.

[59] Mac Arthur A, MacLellan CJ, Malthus T (2012). The fields of view and directional response functions of two field spectroradiometers. IEEE transactions on geoscience and remote sensing.

[60] Rollin EM, Milton EJ, Emery DR (2000). Reference panel anisotropy and diffuse radiation – some implications for field spectroscopy. International Journal of Remote Sensing.

[61] Hueni A, Bialek A (2017). Cause, effect, and correction of field spectroradiometer interchannel radiometric steps. IEEE Journal of Selected Topics in Applied Earth Observations and Remote Sensing.

[62] Geng LY, Ma MG, Yu WP, Wang XF, Jia SZ (2014). Validation of the MODIS NDVI products in different land-use types using *in situ* measurements in the Heihe river basin. IEEE Geoscience and Remote Sensing Letters.

[63] You DQ (2015). Development of a High Resolution BRDF/Albedo Product by Fusing Airborne CASI Reflectance with MODIS Daily Reflectance in the Oasis Area of the Heihe River Basin, China. Remote Sensing.

[64] Wen JG (2017). Forward a Small-Time Scale BRDF/albedo by Multi-sensors Combined BRDF inversion (MCBI) model. IEEE Transactions on Geoscience and Remote Sensing.

[65] Steven RS, John RJ, George TR, Dwayne EP (2004). Temporal Modeling of Bidirectional Reflection Distribution Function (BRDF) in Coastal Vegetation. GIScience & Remote Sensing.

