# Supplementary material for: Full-band, multi-angle, multi-scale, and temporal dynamic field spectral measurements in China
Source: Sci Data. 2023 Jun 3;10:353. doi: 10.1038/s41597-023-02265-1 (PMC10239449; doi:10.1038/s41597-023-02265-1)
Supplement: Supplementary file 1 — Supplementary Table 1 [file 41597_2023_2265_MOESM1_ESM.docx]

| **No.** | **Sample** | **Picture** | **Locality** | **Date** | **Band** |
| --- | --- | --- | --- | --- | --- |
| 1 | ChineseCherry | 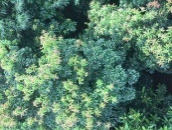 | Guilin | 20170813 | refl_leaf |
|  |  |  |  |  | refl_canopy |
| 2 | ApricotTree | 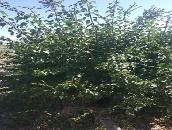 | Zhangye | 20180909 | refl_leaf |
|  |  |  |  |  | refl_canopy |
| 3 | CamelliaJaponica | 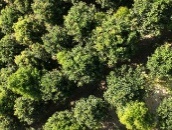 | Chongqin | 20170722 | refl_leaf |
|  |  |  |  |  | refl_canopy |
| 4 | CamelliaOleifera | 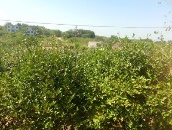 | Jiujiang | 20180904 | refl_leaf |
|  |  |  |  |  | refl_canopy |
| 5 | Clove | 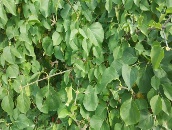 | Baiyin | 20180926 | refl_leaf |
|  |  |  |  |  | refl_canopy |
| 6 | Peony | 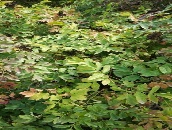 | Baiyin | 20180926 | refl_leaf |
|  |  |  |  |  | refl_canopy |
| 7 | GinkgoBiloba | 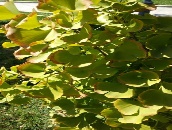 | Baiyin | 20180928 | refl_leaf |
|  |  |  |  |  | refl_canopy |
| 8 | Chrysanthemum | 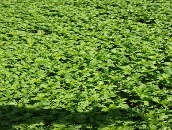 | Baiyin | 20180928 | refl_leaf |
|  |  |  |  |  | refl_canopy |
| 9 | Citrus | 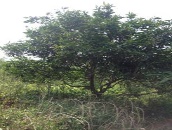 | Chongqin | 20170727 | refl_leaf |
|  |  |  |  |  | refl_canopy |
| 10 | ColonaThorelii | 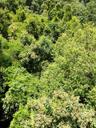 | Xishuangbanna | 20170821 | refl_leaf |
|  |  |  |  |  | refl_canopy |
| 11 | Corn | 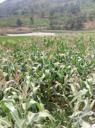 | Chongqin | 20170730 | refl_leaf |
|  |  |  |  |  | refl_canopy |
| 12 | Cotton | 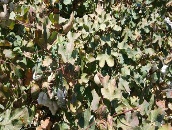 | Jiuquan | 20180915 | refl_leaf |
|  |  |  |  |  | refl_canopy |
| 13 | CyclobalanopsisMyrsinifoliaOersted | 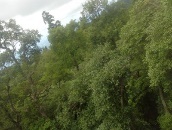 | Hangzhou | 20180830 | refl_leaf |
|  |  |  |  |  | refl_canopy |
| 14 | Cypress | 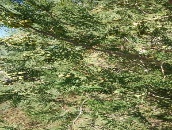 | Jiuquan | 20180915 | refl_leaf |
|  |  |  |  |  | refl_canopy |
| 15 | DaphniphyllumMacropodum | 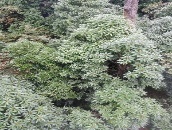 | Hangzhou | 20180830 | refl_leaf |
|  |  |  |  |  | refl_canopy |
| 16 | Edamame | 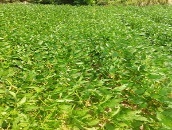 | Jiujiang | 20180904 | refl_leaf |
|  |  |  |  |  | refl_canopy |
| 17 | Eggplant | 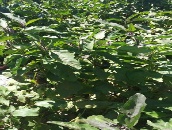 | Zhangjiakou | 20180909 | refl_leaf |
|  |  |  |  |  | refl_canopy |
| 18 | Hawthorn | 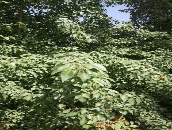 | Zhangye | 20180911 | refl_leaf |
|  |  |  |  |  | refl_canopy |
| 19 | Jujube | 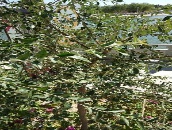 | Jiuquan | 20180915 | refl_leaf |
|  |  |  |  |  | refl_canopy |
| 20 | MaackiaAmurensis | 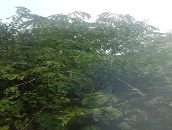 | Lushan | 20180906 | refl_leaf |
|  |  |  |  |  | refl_canopy |
| 21 | Maize | 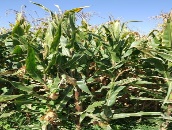 | Baiyin | 20180928 | refl_leaf |
|  |  |  |  |  | refl_canopy |
| 22 | PadusBuergeriana | 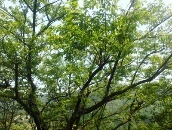 | Lushan | 20180906 | refl_leaf |
|  |  |  |  |  | refl_canopy |
| 23 | PhnomPenhBoxwood | 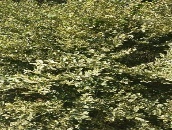 | Xi’an | 20181010 | refl_leaf |
|  |  |  |  |  | refl_canopy |
| 24 | PhyllostachysPraecox | 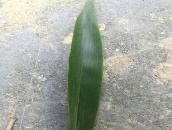 | Hangzhou | 20180830 | refl_leaf |
|  |  |  |  |  | refl_canopy |
| 25 | QuercusGlandulifera | 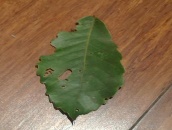 | Hangzhou | 20180830 | refl_leaf |
|  |  |  |  |  | refl_canopy |
| 26 | Rape | 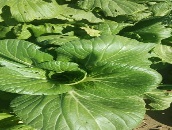 | Jiuquan | 20180915 | refl_leaf |
|  |  |  |  |  | refl_canopy |
| 27 | Reed | 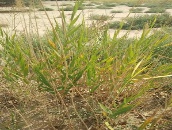 | Hami | 20180917 | refl_leaf |
|  |  |  |  |  | refl_canopy |
| 28 | Rice | 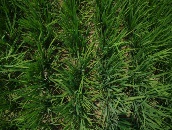 | Xinxiang | 20180726 | refl_leaf |
|  |  |  |  |  | refl_canopy |
| 29 | Sakura | 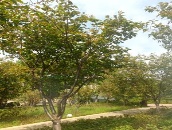 | Changsha | 20170912 | refl_canopy |
|  |  |  |  |  | emis_canopy |
| 30 | soybean | 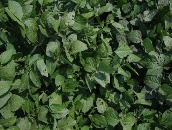 | Xuchan | 20180817 | refl_leaf |
|  |  |  |  |  | refl_canopy |
| 31 | wheat | 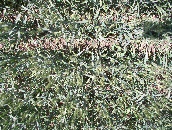 | Beijing | 20040519 | refl_leaf |
|  |  |  |  |  | refl_canopy |

**Table S1.** Information of the multi-scale vegetation reflectance spectra measurement samples.
